# Test-Retest Reliability of the MotionMetrix Software for the Analysis of Walking and Running Gait Parameters

**DOI:** 10.3390/s22093201

**Published:** 2022-04-21

**Authors:** Diego Jaén-Carrillo, Santiago A. Ruiz-Alias, Jose M. Chicano-Gutiérrez, Emilio J. Ruiz-Malagón, Luis E. Roche-Seruendo, Felipe García-Pinillos

**Affiliations:** 1 Faculty of Health Sciences, Campus Universitario, Universidad San Jorge, Autov A23 km 299, Villanueva de Gállego, 50830 Zaragoza, Spain; djaen@usj.es (D.J.-C.); leroche@usj.es (L.E.R.-S.); 2 Department of Physical Education and Sport, University of Granada, 18071 Granada, Spain; aljruiz@ugr.es (S.A.R.-A.); emiliorm@ugr.es (E.J.R.-M.); 3 Sport and Health Research Institute (iMUDS), University of Granada, 18007 Granada, Spain; jchicano@ugr.es; 4 Department of Physical Education, Sports and Recreation, Universidad de La Frontera, Temuco 1145, Chile

**Keywords:** analysis, biomechanics, gait, markerless, testing

## Abstract

The use of markerless motion capture systems is becoming more popular for walking and running analysis given their user-friendliness and their time efficiency but in some cases their validity is uncertain. Here, the test-retest reliability of the MotionMetrix software combined with the use of Kinect sensors is tested with 24 healthy volunteers for walking (at 5 km·h^−1^) and running (at 10 and 15 km·h^−1^) gait analysis in two different trials. All the parameters given by the MotionMetrix software for both walking and running gait analysis are tested in terms of reliability. No significant differences (*p* > 0.05) were found for walking gait parameters between both trials except for the phases of loading response and double support, and the spatiotemporal parameters of step length and step frequency. Additionally, all the parameters exhibit acceptable reliability (CV < 10%) but step width (CV > 10%). When analyzing running gait, although the parameters here tested exhibited different reliability values at 10 km·h^−1^, the system provided reliable measurements for most of the kinematic and kinetic parameters (CV < 10%) when running at 15 km·h^−1^. Overall, the results obtained show that, although some variables must be interpreted with caution, the Kinect + MotionMetrix system may be useful for walking and running gait analysis. Nevertheless, the validity still needs to be determined against a gold standard system to fully trust this technology and software combination.

## 1. Introduction

In both research and diagnosis, the use of marker-based motion capture technologies has been expanded dramatically. However, inherent limits in data collecting may restrict its use in contexts such as patient homes, sports fields, or public spaces where the use of a large number of cameras is impractical. Here, a markerless motion capture system has been offered as one possible solution [1,2].

Markerless systems do not require any markers or sensors to be attached to the body, reducing clinical feasibility and testing time significantly. The lack of markers, on the other hand, may have an impact on measuring accuracy. Thus, investigations examining the validity of such systems under different conditions are crucial. In this context, doctors, sports practitioners, and researchers have been paying close attention to a markerless motion capture system [1,3,4,5,6,7].

The validity of the Kinect™ sensor, created first for interacting with video games on the Microsoft Xbox™ platform by using body movements, for the analysis of gait parameters has been previously evaluated [1,3,4,5,6,7]. Various pieces of software, including various filters and calibrations, have been studied in these works. Whereas Schmitz et al. [1] analyzed the validity of the Kinect™ system with the KinectFusion software for kinematic data assessment, Dolatabadi et al. [3] identified the concurrent validity of the Kinect™ for Windows to measure gait spatiotemporal variables. Then, the validity of the Kinect™ for gait kinematics analysis in comparison with a “gold standard” motion capture system was assessed, operating both systems under Cartesian calibration [4]. Likewise, concurrent validity of the Kinect system for spatiotemporal gait parameters was also assessed [6]. However, none of the mentioned studies considered the MotionMetrix™ software, which might affect measuring accuracy.

As far as the authors’ concern, only one study took into account the Kinect + MotionMetrix combination [8]. Here, the absolute reliability and concurrent validity of the Kinect + MotionMetrix combination was evaluated for spatiotemporal parameters when running at a comfortable velocity by comparing data between the combination system and two widely used systems (i.e., high-speed video analysis and OptoGait). It was found that contact time (CT) was overestimated by the system, whereas flight time (FT) was underrated. However, it resulted to be a valid tool for step frequency (SF) and step length (SL) measures [8]. Although concurrent validity has been assessed, the reliability of the Kinect + MotionMetrix system for either walking or running parameters has not been evaluated.

To identify whether findings are attributable to changes in gait pattern or merely systematic measurement errors, a gait analysis system’s reliability is critical. Therefore, the aim of this study is to analyze the test–retest reliability of both walking and running gait on a treadmill running at 5, 10 and 15 km·h^−1^ by comparing inter-session data obtained from the Kinect + MotionMetrix system.

## 2. Materials and Methods

This study follows the STROBE recommendations for reporting observational studies [9].

### 2.1. Subjects

A group of 16 men and 8 women recreationally active (age = 22.7 ± 2.6 years; body mass = 69.1 ± 11.7 kg; height = 1.72 ± 0.10 m; weekly training = 6.9 ± 2.4 h/week) [10] and familiar with treadmill running voluntarily took part in the study. All of them were free from injuries and reported no physical limitations or health problems. An informed consent was signed by each participant after being informed of the objectives and procedures. It was made clear that they were free to leave at any point. The study followed the Declaration of Helsinki (2013) and was approved by the Ethics Board of the local university (No. 2546/CEIH/2022).

### 2.2. Procedures

Each participant attended the laboratory only once. The participants were instructed to refrain from strenuous activity for, at least, 48 h before data collection [11]. During the test, they wore their usual running clothes and shoes. A treadmill (WOODWAY Pro XL, Woodway, Inc., Waukesha, WI, USA) walking and running protocol was completed. An accommodation period on the treadmill of, at least, 8 min was completed at a self-selected velocity [12]. Thereafter, a protocol where 1-min bouts at 5, 10 and 15 km·h^−1^ was completed. After a 5-min break to avoid fluctuations in the running pattern caused by fatigue, all the participants completed the protocol again. Data were collected during the last 30 s of each bout to guarantee participant adaptation to the running speed

### 2.3. Materials and Testing

Participants body mass (kg) and height (m) were obtained using a bioimpedance scale (Inbody 230, Inbody, Seul, Korea) and a stadiometer (SECA 222, SECA, Corp., Hamburg, Germany), respectively.

**Figure 1:**
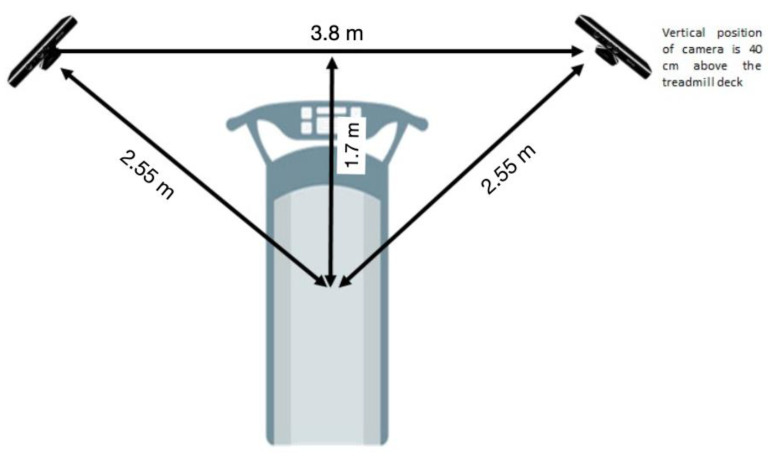
Experimental set-up including treadmill and the positioning of the kinect cameras.

Table 1 shows the definition of all the parameters provided by the MotionMetrix software (MotionMetrix AB). It offers different kinetic and kinematic variables depending on gait velocity used during analysis. The combination Kinect + MotionMetrix was employed to measure such parameters and all the additional variables that the software provides for walking and running gait. To control potential influencing factors for temporal parameters, only the right leg of the participants was analyzed (i.e., asymmetry) [11]. Through the use of a depth sensor, the Microsoft KinectTM sensor (version 1.0, Microsoft, Redmond, WA, USA) can monitor 3-D motions. It can locate 20 body joints in 3D space at 30 Hz. Here, two Microsoft KinectTM sensors were placed on either side of a treadmill in a certain configuration (170 cm from the treadmill’s center in forward direction and 190 cm in the perpendicular direction, according to manufacturer recommendations) and utilized in conjunction with MotionMetrixTM software (Figure 1). The Microsoft KinectTM sensors can reach 60 Hz when both sensors can track the same point at the same time (according to the manufacturer). For data collection, manufacturer recommendations were considered (i.e., software calibration, tight clothes, no shiny black fabric or reflexes, no moving shoelaces, no moving hair, no sunlight, and no treadmill parts blocking the entire view of the participant).

### 2.4. Statistical Analysis

Data are shown as mean, standard deviation (SD), and ranges. Shapiro-Wilk test confirmed the assumption of data normal distribution (*p* > 0.05). A mean comparison analysis (i.e., dependent samples T-test) was applied between variables from both trials of each participant (i.e., test-retest) for magnitude comparison. Cohen’s d effect size (ES) was adopted to interpret the magnitude of the differences following the next criterion: trivial (<0.20), small (0.20–0.59), moderate (0.60–1.19), large (1.20–2.00) and very large (>2.00) [13]. By means of standard error (SE) and coefficient of variation (CV in %, confidence interval (CI): 95%) reliability was assessed [13] and identified as acceptable when CV < 10% [14]. Moreover, intraclass correlation coefficient (ICC, model 3.1) between both trials and for each of the variables analyzed was provided after recommendations by Koo and Li [15]. ICC was interpreted considering the following cut-off values [16]: poor (ICC < 0), trivial (0–0.2), small (0.21–0.40), moderate (0.41–0.60), substantial (0.61–0.80), and almost perfect (>0.81). The 95% CI for these ICCs was also described. Custom spreadsheets were used to assess reliability [17]. The criterion alpha level was set at α = 0.05.

## 3. Results

### 3.1. Test-Retest Reliability and ICC Interpretation during Walking Gait

The test-retest reliability data for the kinematic parameters reported by the Kinect + MotionMetrix system during walking at 5 km·h^−1^ are shown in Table 2.

When analyzing walking gait, significant differences were exhibited only for load response (LR) and double support (DS) phases (*p* = 0.016 and 0.014, respectively) showing a small magnitude of differences (ES = 0.42 and 0.37, respectively) when evaluating both measurements. Moreover, SL and SF showed significant differences (*p* = 0.002 and 0.008, respectively) with a trivial magnitude of differences (ES ≤ 0.12). For the rest of variables, no significant differences were identified.

Walking gait measures showed acceptable reliability for all variables (i.e., CV < 4%) except step width (SW) (CV = 12.12%). The ICC values obtained with markerless system for ST, SL and SF exhibited an almost perfect correlation (ICC = 0.95, 0.94, and 0.96, respectively). Pre-swing phase (PSw), LR, DS and SW showed substantial correlation (ICC < 0.80). Moreover, whereas ICCs for both StP and SwP were interpreted as moderate (<0.60), ICCs for hip frontal angle (HFA) and knee frontal angle (KFA) were considered as poor (ICC < 0).

### 3.2. Test-Retest Reliability and ICC Interpretation during Running Gait

Table 3 and Table 4 show the test-retest reliability data for the kinetic and kinematic variables reported by the Kinect + MotionMetrix system during running at both 10 and 15 km·h^−1^, respectively.

When analyzing running gait at 10 km·h^−1^, significant differences were found between both measures for stride time (StrT), stride length (StrL), SF, SL, maximal thigh flexion (ThighFlex), maximal knee flexion during swing (KFSw), knee rotation (KRot) and SW (*p* < 0.05). The magnitude of the differences for these variables was interpreted as either trivial (ES < 0.20 for SF, ThighFlex, KFSw, KRot, and SW) or small (ES < 0.60 for StrT, StrL, and SL). However, no significant differences for the rest of the variables provided by the system when running at 10 km·h^−1^ (see Table 3).

Table 4 displays running analysis at 15 km·h^−1^ using Kinect + MotionMetrix system. Here, no significant differences between trials were found when analyzing kinematic parameters (*p* > 0.05) except when assessing vertical displacement (Vdisp) and KRot (*p* < 0.03), which also show a small (ES = 0.22) and trivial (ES = 0.17) magnitude of the differences, respectively. When examining kinetic variables, significant differences (*p* < 0.05) were found for vertical force (VertF), maximal loading rate (LRmax), maximal propulsion rate (PRmax), external work (ExW), leg-spring stiffness (LSS), knee vertical force (KFv), knee frontal moment (KMf), hip vertical force (HFv), and hip frontal moment (HMf). The magnitude of the differences for all the parameters mentioned above were identified as small (ES < 0.60) except PRmax, KFv, and HFv that were identified as trivial (ES < 0.20). The rest of the variables show no significant differences when analyzing running gait at 15 km·h^−1^ (see Table 4).

When assessing reliability for running gait analysis at 10 km·h^−1^, all variables seemed to show acceptable reliability (CV < 10%) except when evaluating reliability for foot strike angle (FSA), ankle landing (AL), spine angle (SpA), ThighFlex, knee flexion when landing (KFL), and SW (CV > 10%). The ICCs obtained revealed moderate correlation for thigh extension (ThighExt) and CT (ICC = 0.44 and 0.51, respectively), substantial correlation for ThighFlex, shank angle (ShA), KFL, knee flexion during stance phase (KFS), and knee flexion during swing phase (KFSw) (ICC = 0.67–0.78), and an almost perfect correlation (ICC > 0.81) for the rest of variables at a running speed of 10 km·h^−1^.

Then, when running velocity was set at 15 km·h^−1^, although all the kinematic variables provided by the system show acceptable reliability (CV < 10%), AL, SpA, and SW exhibited CV > 10% for their measures. When kinetic variables were considered, all the parameters show acceptable reliability (CV < 10%) except lateral force (LatF) and elastic exchange EEx showing CV = 13.97% and 21.4%, respectively. The ICC values obtained revealed almost perfect correlation for most of the kinetic and kinematic parameters (ICC > 0.83) except the kinematic parameters of ShA, SpA, and CT (ICC = 0.69, 0.76, 0.80, respectively) and the kinetic parameters of VertF, LRmax, Prmax, KFv, and HFv (ICC = 0.79, 0.71, 0.65, 0.80, and 0.79, respectively). Furthermore, the kinetic variable of elastic exchange (Eex) shows moderate correlation (ICC = 0.58).

## 4. Discussion

This study aimed to determine the test–retest (inter-trial) reliability of the Kinect + MotionMetrix system for the analysis of both walking and running gait parameters (i.e., kinetic and kinematic variables) on a treadmill. Here, twenty-four participants were tested to assess the inter-trial reliability of such markerless system. Our results show that, although there were significant differences between both measurements for both LR and DS phases, and the spatiotemporal parameters of SL and SF, the system seems to provide reliable measurements when analyzing walking gait at 5 km·h^−1^. Then, when considering reliability when running at 10 km·h^−1^, no significant differences were found for most of the variables except when assessing StrT, StrL, SF, SL, ThighFlex, (KFSw), Krot, and SW. The system apparently provides reliable measures for all the variables apart from FSA, AL, SpA, ThighFlex, KFL, and SW. During running at 15 km·h^−1^, no significant differences were found when evaluating kinematic parameters for both trials besides Vdisp and Krot obtaining, additionally, reliable measures from the system for all the kinematic parameters excepting AL, SpA, and SW. If kinematic parameters (i.e., running velocity) are considered, only Vdisp and Krot showed significant differences between both trials, providing the system reliable measurements for all these parameters except when assessing AL, SpA, and SW. For kinetic variables, although the system seems to be reliable when analyzing such parameters (excepting LatF and Eex), significant differences between trials were found for the measures of VertF, Lrmax, Prmax, ExW, LSS, KFv, KMf, HFv, and HMf. The results expose not only the overall intersession reliability of the system when assessing kinematics in walking and running gait, but also its inaccuracy when considering some kinetic parameters.

Research on validity and reliability of markerless motion capture systems for biomechanical analysis during either walking or running on a treadmill is limited. Although the validity of the Kinect™ sensor for walking gait analysis has been assessed [1,3,4,5], the findings reported are controversial. A previous study [3] stated that the Kinect™ sensor used for Windows is a valid tool for measuring walking gait spatiotemporal parameters. Others [5,6] have reported important differences when comparing spatiotemporal parameters measured by the Kinect™ sensor and such parameters by a three-dimensional motion capture system. Particularly, Clark et al. [6] determined that walking gait parameters obtained employing the Kinect™ were lower (i.e., −16% ST, −19% StrT, −1.7% SL) than those acquired utilizing the three-dimensional system. Similarly, Xu et al. [5] claimed that the Kinect™ system reported valid ST and StrT values, but shorter stance time (i.e., −9%) regarding the three-dimensional system when walking. Seemingly, the accuracy of the Kinect™ system in measuring spatiotemporal characteristics is mainly reliant on factors such as the software and filter settings used, the gold standard or reference system examined, or the procedure followed as well as target variables. It is worth noting that the treadmill protocol used in the present study was intended to reduce any potential gait and running variability caused by either treadmill inexperience or fatigue [18,19]. It has been reported that a minimum time of 6 to 8 min is required for healthy young adults and novice treadmill runners to accommodate their locomotion on the treadmill. Thus, it remains unknown whether the Kinect™ system would perform in greater variability conditions.

As treadmill running has been shown to have certain biomechanical variations from running on the ground [12], readers must be cautious when interpreting the results here reported. Some of the investigations that looked into the validity of the Kinect™ system were done on the ground [3,6], whereas just three studies were completed on a treadmill [4,5,7]. This is key as validity or reliability data obtained while walking should not be transferred to running situations since the magnitude of the parameters changes and other phases emerge (i.e., FT does not exist during walking, while there is no double-support time during running). Pfister and colleagues [7] investigated sagittal plane gait kinematics at different walking and running velocities (i.e., 4.8 to 8.8 km·h^−1^), which are lower than the velocity in the current study (i.e., 5, 10 and 15 km·h^−1^), without mentioning kinetic and kinematic parameters, and concluded that the measurement accuracy of the Kinect™ system was not acceptable for clinical measurement analysis (i.e., the system did not provide consistent hip or knee measurements as compared to a three-dimensional system). It is worth mentioning that Pfister [7] employed an older software (i.e., Brekel Kinect) combined with the Kinect™ sensor, which might explain the variations between the studies. The Brekel software operated at 30 Hz, but the software utilized in this study (i.e., MotionMetrix™) can operate at 60 Hz, implying a better level of precision. Indeed, the values obtained in the present study for knee and hip measures present lower CV at the different velocities (<~10%). However, when assessing ankle and spine angles, the CV were greater (>~12%) regardless running velocity.

To the best of the authors’ knowledge, only one study [8] has examined the validity of the Kinect + MotionMetrix system during running. Here, absolute reliability and concurrent validity of this system for measuring CT, FT, SF, and SL was assessed when running at 12 km·h^−1^. It was determined that the Kinect + MotionMetrix system provides valid SF and SL values, but CT and FT are overestimated and underestimated, respectively [8]. Our study complements the aforementioned study by assessing the reliability of the system not only for the spatiotemporal parameters previously mentioned, but for all the parameters (i.e., kinetic and kinematic variables) that the system provides as well. Of note, the MotionMetrix system offers different parameters depending on the walking or running velocity during analysis.

The lack of studies either assessing MotionMetrix™ reliability or employing the system for walking and/or running gait analysis has made the discussion section a challenge, being this the main limitation of the study. At the same time, this study offers evidence-based knowledge to fill such gap and to provide future studies support in the use of Kinect + MotionMetrix system. However, it is worth mentioning that the validity of the kinetics and kinematics variables still needs to be determined against a gold standard system to fully trust this technology and software combination. Furthermore, the sample recruited were active healthy subjects remaining therefore unknown how the system would perform in greater variability conditions such as (i.e., patients with gait disorders)

To sum up, the results indicate that the Kinect + MotionMetrix software provides reliable measures when analyzing walking gait at 5 km·h^−1^ for all the parameters that the software acquires except for SW (CV = 12.12%). Moreover, it provides reliable measurements for all the variables acquired in running at 10 km·h^−1^ except for FSA and AL (CV = 15.90% and 17.46%, respectively), ThighFlex, KFL and SW (CV = 16.26%, 10.14% and 10.72%, respectively). Finally, when running at 15 km·h^−1^, the software also provides reliable values for all the kinematic parameters excepting AL, SpA, and SW (CV = 17.46%, 23.27% and 22.22%, respectively). However, when considering kinetic parameters in running at 15 km·h^−1^, all the acquired values seem to be reliable apart from EEx (CV = 21.40%).

## 5. Conclusions

The results obtained show that, although some variables should be interpreted with caution, the Kinect + MotionMetrix system may be useful for walking and running gait analysis after a simple 30 s calibration. Both researchers and clinicians must be aware of the characteristics of the measures depending on either the walking or running velocity as the reliability of the parameters may fluctuate. The use of the MotionMetrix software offers practitioners a low-cost, time-efficient, and user-friendly motion analysis system for assessing and monitoring both walking and running gait at different velocities. Despite these promising results, the validity of the Kinect + MotionMetrix system still needs to be determined against a gold standard system to fully trust this technology and software combination.

## Figures and Tables

**Table 1:** Definitions of the variables provided by the MotionMetrix software.

**Gait Variables**	**Definition**
Stance phase (% gait cycle)	Period when the foot is in contact with the floor
Swing phase (% gait cycle)	Period when the foot is not in contact with the floor
Load response (ms)	Period of initial double limb support
Pre-swing (ms)	Last phase of stance
Doble support (ms)	Stance with both feet in contact with the floor
Step time (ms)	Interval between initial contacts of the contralateral foot
Step length (cm)	Distance between initial contacts of the contralateral foot
Step frequency (spm)	Step rate per minute
Hip frontal angle (deg)	Hip angle at the coronal plane at the initial single support stage
Knee frontal angle (deg)	Knee angle at the coronal plane at the initial single support stage
Step width (cm)	Distance between the heels of the two feet during double stance
**Running Variables**	**Definition**
Stride time (ms)	Time between initial contacts of the same foot
Stride length (cm)	Distance between initial contacts of the same foot
Step frequency (spm)	Step rate per minute
Step time (ms)	Interval between initial contacts of the contralateral foot
Step length (cm)	Distance between initial contacts of the contralateral foot
Contact time (ms)	Time between initial contact to toe-off
Flight time (ms)	Time between toe off and initial contact of the contralateral foot
Foot strike angle (deg)	Angle between foot and ground at initial contact
Ankle landing (deg)	Angle between foot and shank at initial contact
Center of mass vertical displacement (cm)	Center of mass vertical displacement between steps
Spine angle (deg)	Forward lean
Thigh flexion (deg)	Maximum thigh flexion during the swing phase
Thigh extension (deg)	Maximum thigh extension during the swing phase
Shank angle (deg)	Shank angle at initial contact with respect the vertical axis at a sagittal plane
Landing knee flexion (deg)	Knee flexion at initial contact
Stance knee flexion (deg)	Maximum knee flexion during the stance phase
Swing knee flexion (deg)	Maximum knee flexion during the swing phase
Knee rotation (deg)	Axial rotation of the knee
Step width (cm)	Distance between the heel and the projection of the center of mass
Vertical force (BW)	Maximum vertical force during the stance phase
Brake force (% of max vertical force)	Maximum brake force during the initial contact phase
Lateral force (% of max vertical force)	Maximum lateral force during the stance phase
Maximal loading rate (BW/s)	Speed at which maximum vertical force is achieved
Maximal propulsion rate (BW/s)	Speed at which maximum propulsion force is achieved
External work (Joules/kg/m)	Work done to accelerate the center of mass with respect the environment
Internal work (Joules/kg/m)	Work done to accelerate the body segments with respect the center of mass
Leg-spring stiffness (BW/m)	Vertical leg length in response to the maximum vertical force of a step
Leg length difference at stance phase (cm)	Vertical leg length change during the stance phase
Elastic exchange (%)	Fraction of total work stored and released as elastic energy
Knee mediolateral force (BW)	Maximum medial force at the knee
Knee vertical force (BW)	Maximum vertical force at the knee
Knee frontal moment (BW/m)	Maximum adduction torque at the knee
Knee sagittal moment (BW/m)	Maximum propulsive torque at the knee
Hip mediolateral force (BW)	Maximum medial force at the hip
Hip vertical force (BW)	Maximum vertical force at the hip
Hip frontal moment (BW/m)	Maximum adduction torque at the hip
Hip sagittal moment (BW/m)	Maximum propulsive torque at the hip

ms: milliseconds; cm: centimeters; spm: steps per minute; deg: degrees; BW: bodyweight; %: percentage; BW/s: bodyweight per second; Joules/kg/m: joules per kilogram per meter; BW/m: bodyweight per meter.

**Table 2:** Descriptive data (means, ±SD) and inter-session reliability of kinematic parameters obtained from Kinect + MotionMetrix software walking at 5 km·h^−1^.

Variable	Measure 1 (±SD)	Measure 2 (±SD)	*p*-Value (Cohen’s d)	CV (%) (95% CI)	ICC (95% CI)	Typical Error
StP (% gait cycle)	65.079 (0.67)	65.054 (0.71)	0.849 (−0.04)	0.70 (0.54–0.98)	0.59 (0.25–0.80)	0.45 (0.35–0.64)
SwP (% gait cycle)	34.92 (0.67)	34.94 (0.71)	0.849 (0.04)	1.30 (1.01–1.82)	0.59 (0.25–0.80)	0.45 (0.35–0.64)
LR (ms)	147.72 (8.9)	151.29 (8.3)	0.016 (0.42) *	3.18 (2.47–4.46)	0.71 (0.44–0.86)	4.75 (3.69–6.67)
PSw (ms)	152 (8.9)	153.5 (7.5)	0.287 (0.18)	3.09 (2.40–4.34)	0.69 (0.40–0.85)	4.72 (3.67–6.62)
DS (ms)	299.73 (14.1)	304.83 (13.4)	0.014 (0.37) *	2.20 (1.71–3.09)	0.78 (0.56–0.90)	6.65 (5.17–9.33)
ST (ms)	520.65 (21)	523.25 (23.7)	0.108 (0.12)	1.03 (0.80–1.45)	0.95 (0.88–0.98)	5.39 (4.19–7.56)
SL (cm)	72.68 (3.4)	73.59 (4)	0.002 (0.12) *	1.24 (0.96–1.74)	0.94 (0.88–0.98)	0.74 (0.58–1.04)
SF (spm)	115.1 (4.8)	114.2 (5.4)	0.008 (−0.17) *	0.91 (0.71–1.27)	0.96 (0.92–0.98)	1.04 (0.81–1.46)
HFA (deg)	−0.32 (2.12)	0.18 (2.08)	0.548 (0.24)	-	−0.85 (−0.93–−0.69)	2.85 (2.22–4.00)
KFA (deg)	0.30 (2.16)	−0.62 (2.13)	0.293 (−0.43)	-	−0.92 (−0.97–−0.83)	2.97 (2.31–4.17)
SW (cm)	15.72 (3.92)	16.06 (4.28)	0.545 (0.08)	12.12 (9.42–17.01)	0.79 (0.58–0.91)	1.93 (1.50–2.70)

StT: stance phase; SwT: swing phase; LR: load response; PSw: pre-swing; DS: double support; ST: step time; SL: step length SF: step frequency; HFA: hip frontal angle; KFA: knee frontal angle; SW: step width; ms: milliseconds; spm: steps per minute; deg: degrees; cm: centimeters; SD: Standard deviation; CV: coefficient of variation; %: percentage, ICC: intraclass coefficient; CI: confidence interval; * *p* < 0.05.

**Table 3:** Descriptive data (means, ±SD) and inter-session reliability of the kinematic parameters obtained from Kinect + MotionMetrix software running at 10 km·h^−1^.

Variable	Measure 1 (±SD)	Measure 2 (±SD)	*p*-Value (Cohen’s d)	CV (%) (95% CI)	ICC (95% CI)	Typical Error
StrT (ms)	733.39 (45.0)	744.70 (40.0)	0.023 (0.27) *	2.17 (1.69–3.04)	0.87 (0.72–0.94)	16.04 (12.47–22.50)
StrL (cm)	203.72 (12.5)	206.86 (1.11)	0.023 (0.27) *	2.17 (1.69–3.04)	0.87 (0.72–0.94)	4.46 (3.46–6.25)
SF (spm)	164.23 (10.3)	161.58 (8.71)	0.030 (−0.28) *	2.43 (1.89–3.41)	0.84 (0.67–0.93)	3.96 (3.07–5.55)
ST (ms)	366.69 (22.5)	372.35 (20)	0.023 (0.27) *	2.17 (1.69–3.04)	0.87 (0.72–0.94)	8.02 (6.23–11.25)
SL (cm)	101.86 (6.2)	103.43 (5.6)	0.023 (0.27) *	2.17 (1.69–3.04)	0.87 (0.72–0.94)	2.23 (1.73–3.13)
CT (ms)	279.23 (23.4)	285.23 (21.5)	0.207 (0.27)	5.67 (4.40–7.95)	0.51 (0.14–0.75)	15.99 (12.43–22.44)
FT (ms)	87.46 (30.2)	87.12 (28.6)	0.925 (−0.01)	14 (10.88–19.64)	0.84 (0.66–0.93)	12.22 (9.50–17.14)
FSA (deg)	9.94 (4.1)	9.45 (4.1)	0.276 (−0.12)	15.90 (12.36–22.31)	0.87 (0.72–0.94)	1.54 (1.20–2.16)
AL (deg)	0.066 (0.03)	0.064 (0.03)	0.343 (−0.12)	17.46 (13.57–24.49)	0.84 (0.67–0.93)	0.45 (0.35–0.64)
Vdisp (cm)	8.21 (2.0)	8.46 (1.6)	0.206 (0.13)	7.86 (6.11–11.02)	0.88 (0.75–0.95)	0.55 (0.43–0.77)
SpA (deg)	6.78 (2.2)	6.52 (2)	0.261 (−0.13)	11.98 (9.31–16.81)	0.87 (0.72–0.94)	0.80 (0.62–1.12)
ThighFlex (deg)	24.91 (8.0)	22.38 (6.2)	0.032 (−0.35) *	16.26 (12.64–22.81)	0.73 (0.47–0.87)	3.85 (2.99–5.39)
ThighExt (deg)	−26.42 (4.3)	−27.29 (3.1)	0.306 (−0.23)	-	0.44 (0.05–0.71)	2.85 (2.21–3.99)
ShA (deg)	−5.18 (2.6)	−5.5 (2.8)	0.424 (−0.12)	-	0.76 (0.53–0.89)	1.35 (1.05–1.89)
KFL (deg)	18.93 (3.9)	19.39 (3.6)	0.425 (0.12)	10.14 (7.88–14.23)	0.75 (0.51–0.88)	1.94 (1.51–2.73)
KFS (deg)	44.84 (4.7)	43.88 (4.4)	0.145 (−0.21)	4.97 (3.86–6.97)	0.78 (0.56–0.90)	2.20 (1.71–3.09)
KFSw (deg)	92.98 (16.9)	87.48 (13.2)	0.043 (−0.36) *	9.89 (7.69–13.87)	0.67 (0.38–0.84)	8.92 (6.94–12.52)
KRot (deg)	−0.13 (2.5)	−0.74 (2)	0.037 (−0.27) *	-	0.83 (0.65–0.92)	0.96 (0.75–1.35)
SW (cm)	5.29 (2.1)	4.95 (2.4)	0.047 (−0.15) *	10.72 (8.33–15.04)	0.94 (0.88–0.98)	0.55 (0.43–0.77)

StrT: Stride time; StrL: stride length; SF: step frequency; ST: step time; SL: step length; CT: contact time; FT: flight time; FSA: foot strike angle; AL: ankle landing; Vdisp: vertical displacement of the center of mass; SpA: spine angle; ThighFlex: thigh flexion; thighExt: thigh extension; ShA: Shank angle; KFL: knee flexion when landing; KFS: knee flexion stance; KFSw: knee flexion swing; Krot: knee rotation; SW: step width; spm: steps per minute; cm: centimeters; deg: degrees; CV: coefficient of variation; %: percentage, ICC: intraclass coefficient; CI: confidence interval. * *p* < 0.05.

**Table 4:** Descriptive data (means, ±SD) and inter-session reliability of the kinematic and kinetic parameters obtained from Kinect + MotionMetrix software running at 15 km·h^−1^.

	Variable	Measure 1 (±SD)	Measure 2 (±SD)	*p*-Value (Cohen’s d)	CV (%) (95% CI)	ICC (95% CI)	Typical Error
Kinematics	StrT (ms)	668.97 (47.2)	675.02 (42.4)	0.051 (0.13)	1.51 (1.18–2.12)	0.95 (0.89–0.98)	10.18 (7.91–14.28)
	StrL (cm)	281.74 (19.7)	281.26 (17.7)	0.051 (0.13)	1.51 (1.18–2.12)	0.95 (0.89–0.98)	4.24 (3.30–5.95)
	SF (spm)	179.43 (11.2)	178.43 (11)	0.223 (−0.09)	1.55 (1.20–2.17)	0.94 (0.87–0.97)	2.77 (2.15–3.89)
	ST (ms)	335.69 (21.8)	337.51 (21.2)	0.224 (0.08)	1.50 (1.16–2.10)	0.95 (0.89–0.98)	5.04 (3.92–7.07)
	SL (cm)	139.87 (9.1)	140.63 (8.8)	0.224 (0.08)	1.50 (1.16–2.10)	0.95 (0.89–0.98)	2.10 (1.63–2.95)
	CT (ms)	223.83 (11.4)	223.14 (11.8)	0.657 (−0.06)	2.38 (1.85–3.34)	0.80 (0.60–0.91)	5.32 (4.14–7.47)
	FT (ms)	111.86 (23.2)	114.37 (22.6)	0.250 (0.11)	6.52 (5.06–9.14)	0.90 (0.79–0.96)	7.37 (5.73–10.34)
	FSA (deg)	12.99 (5.5)	13.11 (5.2)	0.680 (0.02)	7.52 (5.85–10.55)	0.97 (0.93–0.99)	0.98 (0.76–1.38)
	AL (deg)	0.067 (0.03)	0.063 (0.03)	0.343 (−0.12)	17.46 (13.57–24.49)	0.84 (0.67–0.93)	0.01 (0.01–0.02)
	Vdisp (cm)	7.18 (1.9)	7.58 (1.7)	0.029 (0.22) *	8.09 (6.29–11.35)	0.90 (0.78–0.96)	0.60 (0.46–0.84)
	SpA (deg)	6.6 (3.3)	7.14 (3)	0.257 (0.17)	23.27 (18.09–32.64)	0.76 (0.52–0.89)	1.60 (1.24–2.24)
	ThighFlex (deg)	33.93 (5.97)	33.3 (6.28)	0.177 (−0.10)	4.72 (3.67–6.62)	0.94 (0.86–0.97)	1.59 (1.23–2.23)
	ThighExt (deg)	−35.35 (3.66)	−36.19 (2.91)	0.4 (−0.25)	-	0.85 (0.69–0.93)	1.33 (1.03–1.86)
	ShA (deg)	−0.5 (2.6)	−1.4 (2.8)	0.057 (−0.33)	-	0.69 (0.40–0.85)	1.54 (1.20–2.17)
	KFL (deg)	17.02 (4.07)	17.53 (4.06)	0.241 (0.13)	8.51 (6.62–11.94)	0.88 (0.74–0.95)	1.47 (1.14–2.06)
	KFS (deg)	40.9 (4.5)	40.6 (4.3)	0.35 (−0.07)	2.84 (2.21–3.98)	0.94 (0.86–0.97)	1.16 (0.90–1.62)
	KFSw (deg)	113.16 (11.5)	113.24 (10.36)	0.93 (0.01)	2.74 (2.13–3.84)	0.93 (0.84–0.97)	3.10 (2.41–4.35)
	Krot (deg)	−0.07 (2.6)	0.35 (2.4)	0.01 (0.17) *	-	0.96 (0.91–0.98)	0.53 (0.41–0.74)
	SW (cm)	4.39 (2.98)	4.43 (2.65)	0.88 (0.02)	22.22 (17.27–31.17)	0.89 (0.76–0.95)	0.98 (0.76–1.38)
Kinetics	VertF (BW)	2.44 (0.3)	2.52 (0.2)	0.017 (0.35) *	4.78 (3.71–6.70)	0.79 (0.58–0.91)	0.12 (0.09–0.17)
	BrakeF (Fv)	0.112 (0.02)	0.114 (0.03)	0.319 (0.10)	7.56 (5.88–10.61)	0.89 (0.77–0.95)	0.01 (0.01–0.01)
	Lrmax (BW/s)	30.04 (4.7)	31.98 (4.3)	0.013 (0.43) *	8.07 (6.27–11.31)	0.71 (0.44–0.86)	2.50 (1.94–3.51)
	Prmax (BW/s)	−27.14 (4.6)	−29.08 (3.9)	0.017 (−0.45) *	-	0.65 (0.34–0.83)	2.60 (2.02–3.65)
	ExW (Joules/kg/m)	0.42 (0.1)	0.44 (0.1)	0.034 (0.22) *	8.68 (6.75–12–17)	0.89 (0.77–0.95)	0.04 (0.03–0.05)
	IntW (Joules/kg/m)	0.69 (0.08)	0.7 (0.07)	0.58 (0.05)	3.05 (2.37–4.28)	0.92 (0.83–0.97)	0.02 (0.02–0.03)
	ExWg (Joules/kg/m)	1.12 (0.28)	1.17 (0.25)	0.079 (0.2)	8.58 (6.62–11.95)	0.87 (0.73–0.94)	0.1 (0.08–0.14)
	LSS (BW/m)	52.91 (10)	56.79 (11.8)	0.007 (0.35) *	8.31 (6.46–11.65)	0.84 (0.66–0.93)	4.56 (3.54–6.39)
	ΔLegSt (cm)	4.5 (0.9)	4.4 (0.9)	0.23 (0.11)	7.02 (5.46–9.85)	0.91 (0.80 −0.96)	0.31 (0.24–0.44)
	LatF (Fv)	0.0337 (0.01)	0.0331 (0.01)	0.62 (−0.05)	13.97 (10.86–19.6)	0.87 (0.73–0.94)	0.00 (0.00–0.01)
	EEx (%)	31.06 (11.9)	31.32 (8)	0.89 (0.03)	21.40 (16.63–30.01)	0.58 (0.25–0.80)	6.67 (5.19–9.36)
	KFm (BW)	−0.092 (0.03)	−0.093 (0.03)	0.53 (−0.06)	-	0.90 (0.79–0.96)	0.01 (0.01–0.01)
	KFv (BW)	−2.29 (0.25)	−2.38 (0.22)	0.01 (−0.38) *	-	0.80 (0.58–0.91)	0.11 (0.09–0.15)
	KMf (BW/m)	0.12 (0.02)	0.13 (0.02)	0.002 (0.32) *	6.02 (4.68–8.44)	0.91 (0.80–0.96)	0.01 (0.01–0.01)
	KMs (BW/m)	0.41 (0.08)	0.42 (0.08)	0.33 (0.11)	7.11 (5.53–9.98)	0.87 (0.72–0.94)	0.03 (0.02–0.04)
	HFm (BW)	−0.097 (0.03)	−0.097 (0.03)	0.95 (−0.01)	-	0.90 (0.78–0.96)	0.01 (0.01–0.01)
	HFv (BW)	−2.07 (0.24)	−2.15 (0.20)	0.01 (−0.38) *	-	0.79 (0.58–0.90)	0.10 (0.08–0.15)
	HMf (BW/m)	0.24 (0.03)	0.25 (0.03)	0.016 (0.28) *	4.42 (3.44–6.2)	0.87 (0.73–0.94)	0.01 (0.01–0.02)
	HMs (BW/m)	0.54 (0.09)	0.55 (0.09)	0.34 (0.12)	7.07 (5.5–9.9)	0.84 (0.66–0.93)	0.04 (0.03–0.05)

StrT: stride time; StrL: stride length; SF: step frequency; ST: step time; SL: step length; CT: contact time; FT: flight time; FSA: foot strike angle; AL: ankle landing; Vdisp: vertical displacement of the center of mass; SA: spine angle; ThighFlex: thigh flexion; thighExt: thigh extension; ShA: shank angle; KFL: knee flexion landing; KFS: knee flexion stance; KFSw: knee flexion swing; KRot: knee rotation; SW: step width; VertF: vertical force; VertImp: vertical impulse; BrakeF: brake force; LRmax: maximal loading rate; PRmax: maximal propulsion rate; ExW: velocity-normalized external work; IntW: internal work; ExWg: gravity-normalized external work; LSS: leg-spring stiffness; ΔLegSt: leg length difference at stance phase; LatF: lateral force; EEx: elastic exchange (i.e., fraction of total work stored and released as “free” elastic energy in muscle and tendons); KFm: knee mediolateral force; KFv: knee vertical force; KMf: knee frontal moment; KMs: knee sagittal moment; HFm: hip mediolateral force; HFv: hip vertical force; HMf: hip frontal moment; HMs: hip sagittal moment; spm: steps per minute; cm: centimeters; deg: degrees; BW: body weight; BW/m: body weight per meter; CV: coefficient of variation; %: percentage, ICC: intraclass coefficient; CI: confidence interval. * *p* < 0.05.

## Data Availability

The data presented in this study are available on request from the corresponding author. The data are not publicly available due to the participants privacy.
